# First assistant experience in total laparoscopic pancreaticoduodenectomy: accelerating the learning curve for an operator

**DOI:** 10.1186/s12893-023-01987-8

**Published:** 2023-04-17

**Authors:** Dongrui Li, Chengxu Du, Wenbin Wang, Jiansheng Zhang, Jianhua Liu

**Affiliations:** Department of Hepatobiliary Surgery, the Second Hospital of Hebei Medical University, 215 Heping West Road, Shijiazhuang, 050000 Hebei China

**Keywords:** FAE, Total laparoscopic pancreaticoduodenectomy, Learning curve, CUSUM

## Abstract

**Objective:**

Compare and analyze clinical data of total laparoscopic pancreaticoduodenectomy (TLPD) cases for surgeons with / without first assistant experience (FAE) in TLPD. Probe influence of FAE in TLPD on the learning curve for an operator.

**Methods:**

The clinical data of 239 patients, that underwent TLPD performed by two surgeons between January 2017 and January 2022) in our department, were consecutively collected and divided into two groups (A and B). Group A cases were operated by Surgeon A, with FAE of 57 TLPDs in our department prior to initial TLPD as an operator. Group B cases were operated by Surgeon B with no FAE of TLPD. Cumulative sum (CUSUM) method developed learning curves. Clinical data and both surgeons’ learning curves were statistically compared between both groups.

**Results:**

Between both groups, no statistically significant variations were observed for pre-operative health conditions. Reduced surgical duration, blood loss and transfusion volume during surgery, together with reductions in major post–operative complication rates and reduced hospital/ICU stays were identified within Group A, having statistically significant variations. The technical plateau phases of the learning curves were approximately 25–41 cases and 35–51 cases, for Surgeon A and Surgeon B, respectively.

**Conclusion:**

FAE in TLPD can accelerate the learning curve of TLPD for an operator, with safer surgical procedures and enhanced post–operative recovery.

## Introduction

Presently, total laparoscopic pancreaticoduodenectomy (TLPD) is a technically demanding and well-established procedure, with similar or enhanced short-term outcomes and long-term survival rates in comparison to open laparoscopic pancreaticoduodenectomy (OPD) in large-volume centers [1, 2]. Abundant experience of OPD, excelling laparoscopic surgical skills and learning curve are required to ensure the feasibility and safety of LPD [3–6]. To better adopt LPD in medical centers, there have been many studies concerning the learning curve for LPD, using differing sample sizes. Three phases (initial learning period, technical plateau period and technical proficiency period), with two peaks, were typically revealed by analyses of such learning curves. Studies on over 50 cases suggested that the operator could achieve technical competence in TLPD after successfully accomplishing 40–50 TLPD cases [7–18]. However, there are few comparative studies focusing on how to safely and efficiently accelerate the learning curve for TLPD within an operator. This study comparatively analyzed clinical data of TLPD cases and related parameters for learning curves in surgeons with / without First Assistant Experience (FAE), aiming at elucidating the influence of FAE in TLPD on the learning curve for an operator.

## Methods and materials

### Clinical data collection

Clinical data from 239 TLPD cases - subsequently performed by two junior attending surgeons (Surgeon A and B) under identical mentorship throughout the entire surgical procedure, in our department between January 2017 and January 2022 - were collected. All surgeons employed identical laparoscope platform (Olympus™ 3D laparoscopy), with identical assistant / laparoscope handler. All 239 cases were clearly diagnosed as a tumor(< 2 cm) within pancreatic head, distal bile duct, duodenal or ampulla -identified through imaging, though excluding the possibility of inflammatory mass presence. Inclusion criteria were: (1) standard TLPD with no hand-assisting or transversion to OPD; (2) no metastasis in other organs or artery / vein invasion.

The cases were divided into Group A (the first 127 consecutive TLPD cases performed by Surgeon A) and Group B (the first 112 consecutive TLPD cases performed by Surgeon B) based upon differing operators. Both surgeons were second-generation TLPD operators in our department. Prior to the initial TLPD case as an operator, Surgeon A had participated in 57 TLPD cases in our department as First Assistant, while surgeon B had no FAE. Both surgeons had a minimum of 6-years’ experience and were competent of fundamental laparotomic and laparoscopic hepatobiliary-pancreatic surgical procedures, including cholecystectomy, bile duct exploration, chole-jejunostomy, hepatectomy, distal pancreatectomy. Furthermore, both surgeons had a FAE of approximately 30 OPD cases, though had rare experience as an OPD operator. Pre-operative data included general conditions (age, sex, BMI and American Society of Anesthesiologists (ASA) score), initial symptoms (jaundice, abdominal pain and fever), co-morbidities (coronary heart disease (CHD), hypertension (HT), diabetes mellitus (DM), pancreatitis, hepatitis and surgical procedure history), blood test results (CA19-9, CA12-5 and direct bilirubin) and pre-operative treatments alleviating jaundice (percutaneous trans-hepatic cholangial drainage (PTCD) and endoscopic naso-biliary drainage (ENBD)). Intra-operative data included pancreatic texture, size of main pancreatic duct, blood loss, intra-operative transfusion, transfusion volume, operation time, pancreatojejunostomy time, choledochojejunostomy time and gastrojejunostomy time. Post-operative data included post-operative complications (post-operative pancreatic fistula, delayed gastric emptying, post-pancreatectomy hemorrhage and abdominal infection), Clavien-Dindo classification of surgical complications, pathology results such as tumor locations (duodenum, ampulla, distal bile duct and pancreatic head), resection margin status and number of harvested lymph nodes, re-operation, hospitalization time, ICU stay time and mortality event within hospital.

Post-operative pancreatic fistula (POPF) [19], post-pancreatectomy hemorrhage (PPH) [20], delayed gastric emptying [21] and Clavien-Dindo classification of surgical complications [22] were defined according to known consensus and guidelines. Abdominal infection was defined as post-operative fever and increased level of white blood cells (> 10*10^9/L), with exclusion of infections in other organs. Operation time was defined as the duration from the first incision up to final closure; anastomosis time was defined as the duration from finishing time of the former procedure to the completing time for anastomosis procedure; resection time was deemed as the operation time without the anastomosis time.

### TLPD procedure protocol outline

The patient was lying in a supine position with legs placed apart, whereby the operator and assistant were positioned on the right side and left side of the patient, respectively, while laparoscope handler stood between the patient’s legs. Five trocars were inserted in a “U” shape. Theses consisted of one 12 mm trocar at the lower edge of the umbilicus, as the laparoscope port, while one 5 mm trocar, one 12 mm trocar, one 5 mm trocar and one 10 mm trocar were situated at the left and right anterior axillary line below costal margin, left and right midclavicular line above the umbilicus, respectively. The abdominal cavity was filled with carbon dioxide gas at a pressure of 12-14mmHg.

Resection: (1) Dissect gastrocolic ligament and mobilize duodenum through Kocher maneuver. (2) Dissect, ligate and transect the right gastroepiploic and pancreaticoduodenal inferior vessels. (3) Transect the distal stomach 2–3 cm from the pylorus. (4) Expose the jejunum through the Riolan avascular area (on the left to the SMV) and transect the jejunum 15–20 cm distal from the Treitz ligament. (5) Complete lymphadenectomy of hepatoduodenal ligament and transect the gastroduodenal artery. (6) Totally mobilize the jejunum and duodenum from left to right, to expose major vasculatures. (7) Create a tunnel between the pancreatic neck and the superior mesenteric vein (SMV) or portal vein (PV), at the inferior border of the pancreas. (8) Transect pancreatic neck. (9) Complete cholecystectomy and transect common hepatic duct. (10) Expose inferior vena cava (IVC) and left renal vein through Kocher maneuver. Separate the uncinate process from SMV. 11. Complete lymphadenectomy, including lymph node stations 5, 6, 8, 12, 13a, 13b, 14a, 14b, 17a and 17b. 12. Samples placed in a retrieval bag and extracted through a 5-cm upper abdominal incision.

Reconstruction: (1) Pancreatojejunostomy: a two-layer duct-to-mucosa anastomosis. (2) Choledochojejunostomy: an end-to-side anastomosis with approximately 10 cm distance distal to anastomosis for pancreatojejunostomy. (3) Gastrojejunostomy: antecolic gastrojejunostomy 40–45 cm downstream from choledochojejunostomy location.

The principles of uncinate-process-first, novel-artery-first and inferoposterior duodenal approaches were followed during such TLPD procedures. Ultrasonic Shears and linear staplers were used in such procedures, while vessels were ligated with hemlock clips.

The principles of postoperative management for TLPD were as follows: 1. Postoperative infusion treatment include anti-infection, gastric acid suppression, liver function protection, reduce phlegm and nutritional support. 2.The gastric tube remained in place until the patient passed flatus, and a fluid-based diet was administered to the patient once gastric tube was removed. 3. The two drainage tubes were regularly removed sequentially, two days post-operation, once there was no obvious drainage or clear drainage-fluid less than 50mL/day without POPF; the drainage tubes remained if the drainage-fluid was turbid, > 50mL/day or with POPF. 4. Besides the emergency reoperations, laparoscopic reoperations for debridement were performed when the patients had deteriorating abdominal infection and POPF within one week post-LPD.

### Statistical analysis

Clinical data were statistically described and compared between Group A and Group B. Normality tests were performed for quantitative data. Independent-samples’ t tests were performed for parameters such as age, BMI and operation time. Wilcoxon rank sum tests were performed for parameters such as blood test results, pancreatojejunostomy time, choledochojejunostomy time, gastrojejunostomy time, intra-operative blood loss and transfusion volume, ASA score, POPF, hospitalization time and ICU stay duration. Chi-square tests were performed for parameters such as sex, initial symptoms, co-morbidities, pre-operative treatments, intra-operative transfusion, complications (except for POPF), Clavien-Dindo classification, reoperation, pathology results and mortality event in hospital.

The learning curves for both surgeons were developed respectively with cumulative sum (CUSUM) method, which was a graphical method detecting data trends. Cases were ordered chronologically, based upon TLPD duration. CUSUM for operation time was determined as:

$$ CUSUM_{\text{OT}} = {\sum}_{\text{i}=1}^{\text{n}}\left(\text{x}\text{i}-{\mu }\right)$$

where $\text{x}\text{i}$ is the operation time of case i and ${\mu }$ is the mean operation time. CUSUM of pancreatojejunostomy time (CUSUM_PJT_), choledochojejunostomy time (CUSUM_CJT_) and gastrojejunostomy time (CUSUM_GJT_) were also determined through the method applied as for CUSUM_OT_. Differing phases were defined according to CUSUM_OT_ learning curve.

All tests were two-tailed, with P values < 0.05 deemed to confer statistical significance. Statistical analyses were performed by the SPSS for Windows® statistical package.

This study was performed in accordance with relevant guidelines and regulations, and was approved by the Research Ethics Committee of the Second Hospital of Hebei Medical University (No. 2019-R209).

## Results

### Statistical characteristics and comparative analyses

There were no statistical variations in pre-operative data. The statistical characteristics and results of such comparison tests for pre-operative data are described in Table 1.

**Table 1:** Statistical characteristics and results of the comparison tests of preoperative data

Parameter	Group A(127 cases)	Group B(112 cases)	P
General conditions			
Age(years)	60.27 ± 9.78	59.88 ± 9.49	0.832^#^
Sex (male/female)	78/49	61/51	0.277^*^
BMI(kg/m^2^)	22.58 ± 2.91	24.28 ± 3.45	0.083^#^
ASA(n,I/II/III/IV)	2/101/23/1	0/94/18/0	0.402^*^
Initial symptoms [n (%)]			
Jaundice	74(58.27%)	67(59.82%)	0.807^*^
Abdominal pain	54(42.51%)	49(43.75%)	0.848^*^
Fever	12(9.44%)	4(3.57%)	0.070^*^
Comorbidities [n (%)]			
CHD	7(5.51%)	10(8.92%)	0.305^*^
HBP	39(30.70%)	40(35.71%)	0.412^*^
DM	20(15.75%)	16(14.28%)	0.752^*^
Pancreatitis history	4(3.15%)	7(6.25%)	0.254^*^
Hepatitis	8(6.30%)	8(7.14%)	0.928^*^
Opearation history	13(10.24%)	15(13.39%)	0.449^*^
Blood tests			
CA199(U/mL)^a^	94.50	68.50	0.315^+^
CA125(U/mL)^a^	12.90	14.70	0.323^+^
Direct bilirubin(mmol/L)^a^	60.00	64.00	0.773^+^
Preoperative treatments [n (%)]			
Preoperative PTCD	46(36.22%)	36(32.14%)	0.508^*^
Preoperative ENBD	17(13.38%)	13(11.60%)	0.679^*^

BMI: body mass index; ASA: American Society of Anesthesiologists CHD: coronary heart disease; HBP: high blood pressure; DM: diabetes mellitus; PTCD: percutaneous tranhepatic cholangial drainage; ENBD: endoscopic nasalbiliary drainage.

a: Median of the parameter; #: Independent-samples t tests; *: Chi-square tests; +: Wilcoxon rank sum tests

No Statistically significant variations were observed across pancreatic texture and main pancreatic duct (MPD) size between both groups. There were statistical variations in all remaining intra-operative data between Group A and Group B. Such variations suggested that there was reduced blood loss (400mL (median) in Group A and B accordingly, P < 0.001) and transfusion volume (0mL (median) in Group A and 400mL (median) in Group B, P < 0.001), lower intra-operative transfusion rate (44.88% in Group A and 78.57% in Group B, P < 0.001), and reduced operation time (424.41 ± 91.41 min in Group A and 449.11 ± 76.29 min in Group B, P < 0.001), pancreatojejunostomy time (40 min (median) in Group A and 50 min (median) in Group B, P < 0.001), choledochojejunostomy time (20 min (median) in Group A and 30 min (median) in Group B, P < 0.001) and gastrojejunostomy time (15 min (median) in Group A and 25 min (median) in Group B, P < 0.001), in Group A - when compared to Group B - with statistical significance. There were no statistically significant variations in resection time. Among post-operative data, there were statistical variations in major post-operative complications rates (11.02% in Group A and 30.36% in Group B, P < 0.001), hospitalization time (15 days (median) in Group A and 19days (median) in Group B, P < 0.001) and ICU stay duration (0 days (median) in Group A and 0 days (median) in Group B, P < 0.001). Minimized major post-operative complications rates, reduced hospital / ICU stay durations together with increased quantities of harvested lymph nodes were found in Group A. There were no statistically significant variations in tumor location and resection margin status between both groups. The reoperation rate was 5.51% and 12.50%, in Group A and Group B, respectively with no statistical variation. Hospital-based mortality rates were 3.15% and 3.57% in Group A and Group B, accordingly, with no statistical variations. Safer and more efficient surgical procedures, together with enhanced post-operative recovery, were demonstrated in Group A. Statistical characteristics and results for comparative analyses of intra-/post–operative data are described in Table 2.

**Table 2:** Statistical characteristics and results of the comparison tests of intra/post-operative data

Parameter		Group A(127 cases)		Group B(112 cases)		P
Intraoperative						
Pancreatic texture [n (%)]						0.576*
Soft pancreas		113(88.98%)		97(86.61%)		
Not-soft pancreas		14(11.02%)		15(13.39%)		
MPD size(cm) ^a^		0.3		0.3		0.651^+^
Blood loss(mL)^a^		400		500		< 0.001^+^
Transfusion [n (%)]		57(44.88%)		88(78.57%)		< 0.001^*^
Transfusion volume(mL)^a^		0		400		< 0.001^+^
Operation time(min)		424.41 ± 91.41		449.11 ± 76.29		0.020^#^
Pancreatojejunostomy time(min)^a^		40.00		50.00		< 0.001^+^
Choledochojejunostomy time(min)^a^		20.00		30.00		< 0.001^+^
Gastrojejunostomy time(min)^a^		15.00		25.00		< 0.001^+^
Resection time(min)		347.44 ± 84.22		345.94 ± 71.54		0.883^#^
Complications [n (%)]						
Pancreatic fistula (BL/A/B/C)		96(75.59%)/2/11/7		81(72.32%)/7/11/3		0.311^+^
Delayed gastric emptying		3(2.36%)		6(5.35%)		0.225^*^
Postoperative hemorrhage		9(7.09%)		15(16.07%)		0.106^*^
Abdominal infection		13(10.24%)		20(17.86%)		0.088^*^
Clavien-Dindo classification [n (%)] Minor(Grade I and II) Major(Grade III to V)		36(28.35%) 14(11.02%)		45(40.18%) 34(30.36%)		< 0.001^*^
Reoperation [n (%)]		7(5.51%)		14(12.50%)		0.057^*^
Death in hospital [n (%)]		4(3.15%)		4(3.57%)		0.856^*^
Hospital stay (day)^a^		15		19		< 0.001^+^
ICU stay (day)^a^		0		0		< 0.001^+^
Pathological results						
Tumor Location [n (%)]						0.132^*^
Pancreatic head tumor		42(33.07%)		51(45.53%)		
Bile duct tumor		44(34.64%)		28(25.00%)		
Duodenal tumor		31(24.41%)		21(18.75%)		
Ampulla tumor		10(7.87%)		12(10.71%)		
Harvested lymph nodes^a^		12(10,14)		6(2,8)		< 0.001^+^
Resection margin (positive/negative)		4(3.15%)/123(96.85%)		7(6.25%)/105(93.75%)		0.254^*^

a: Median of the parameter; #: Independent-samples t tests; *: Chi-square tests; +: Wilcoxon rank sum tests.

### Learning curves with CUSUM method

**Fig. 1:**
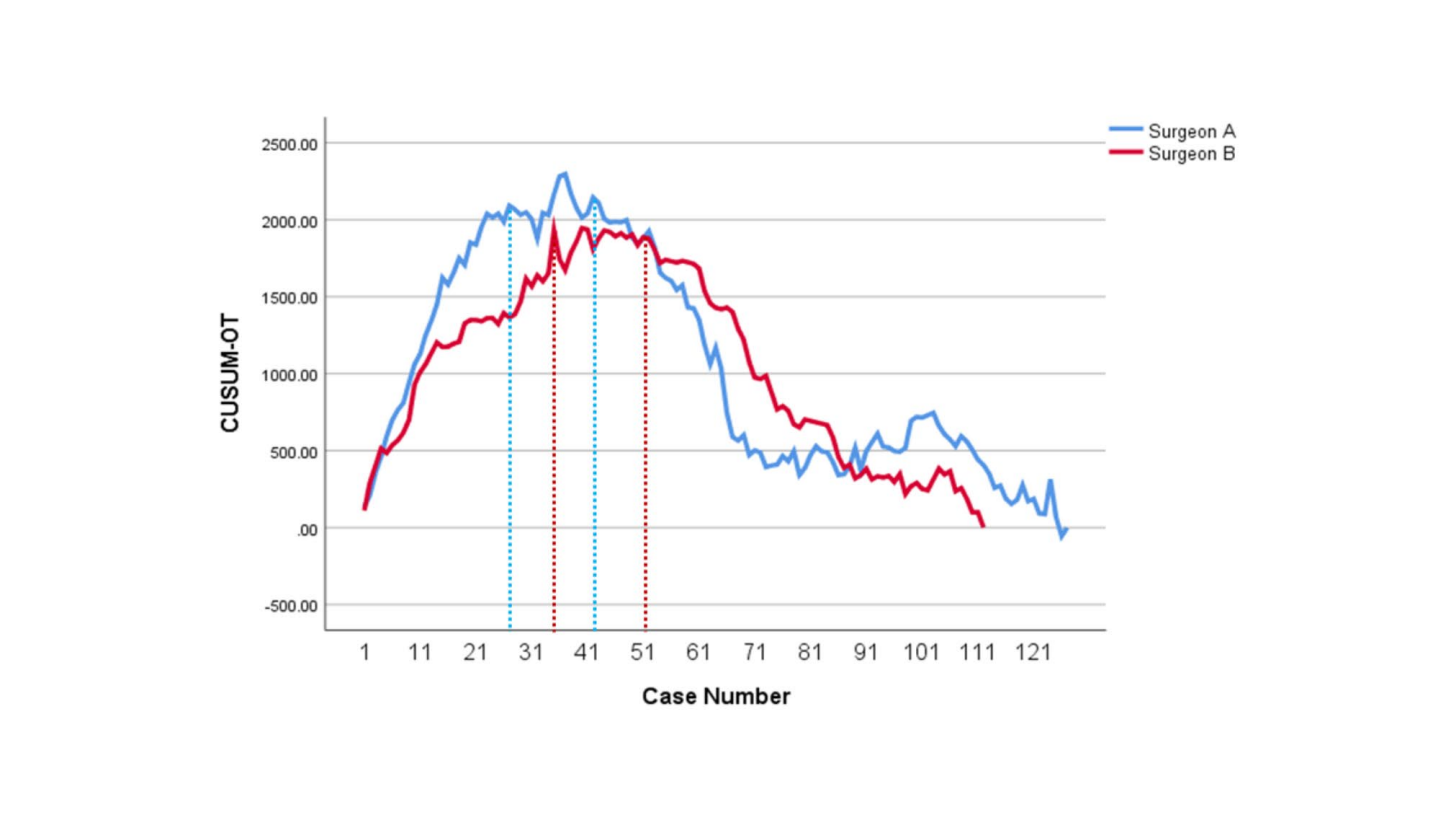
Cumulative sum graphs for operation time (CUSUMOT) of Surgeon A and Surgeon B

**Fig. 2:**
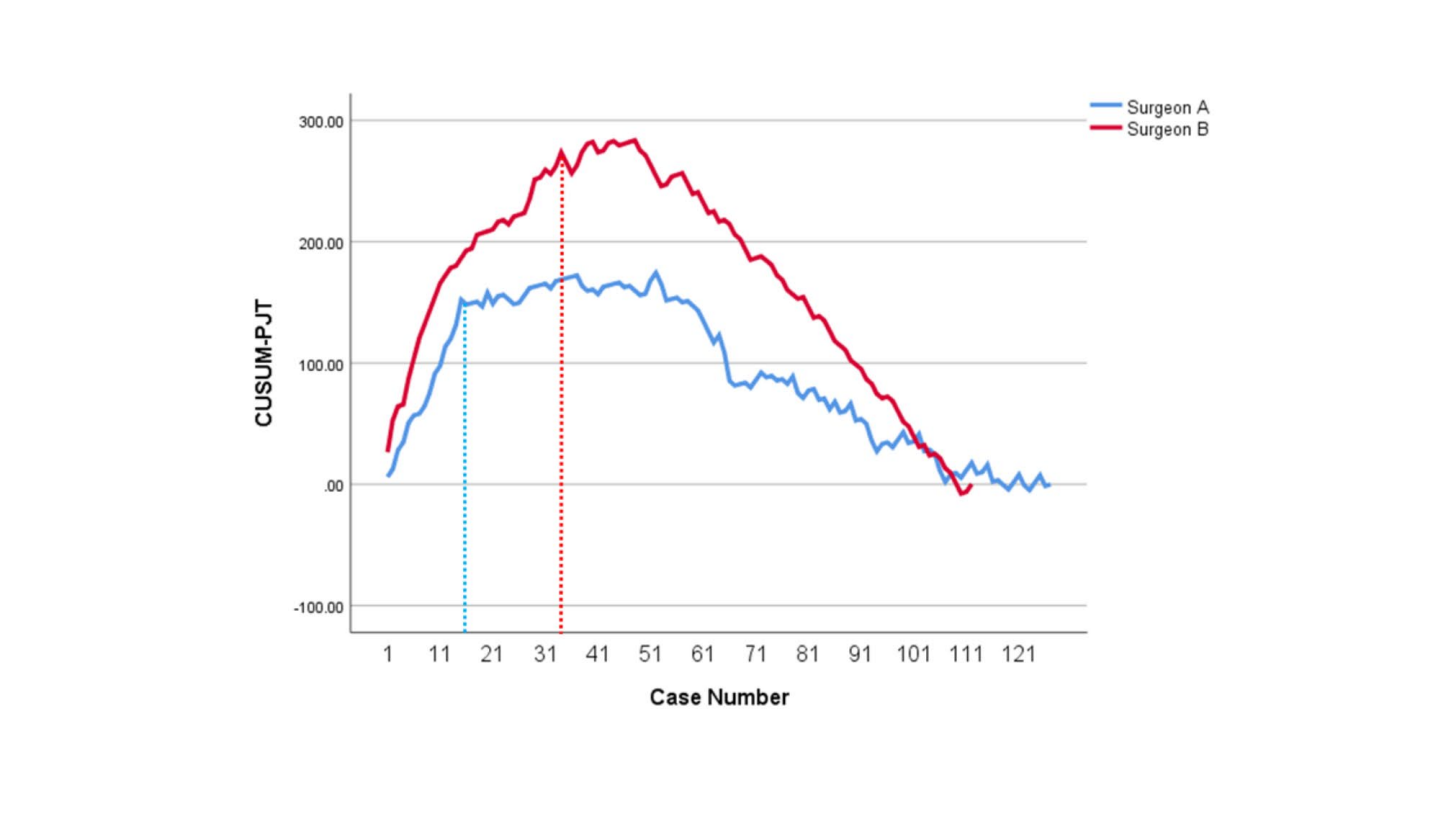
Cumulative sum graphs for pancreatojejunostomy time (CUSUMPJT) of Surgeon A and Surgeon B

**Fig. 3:**
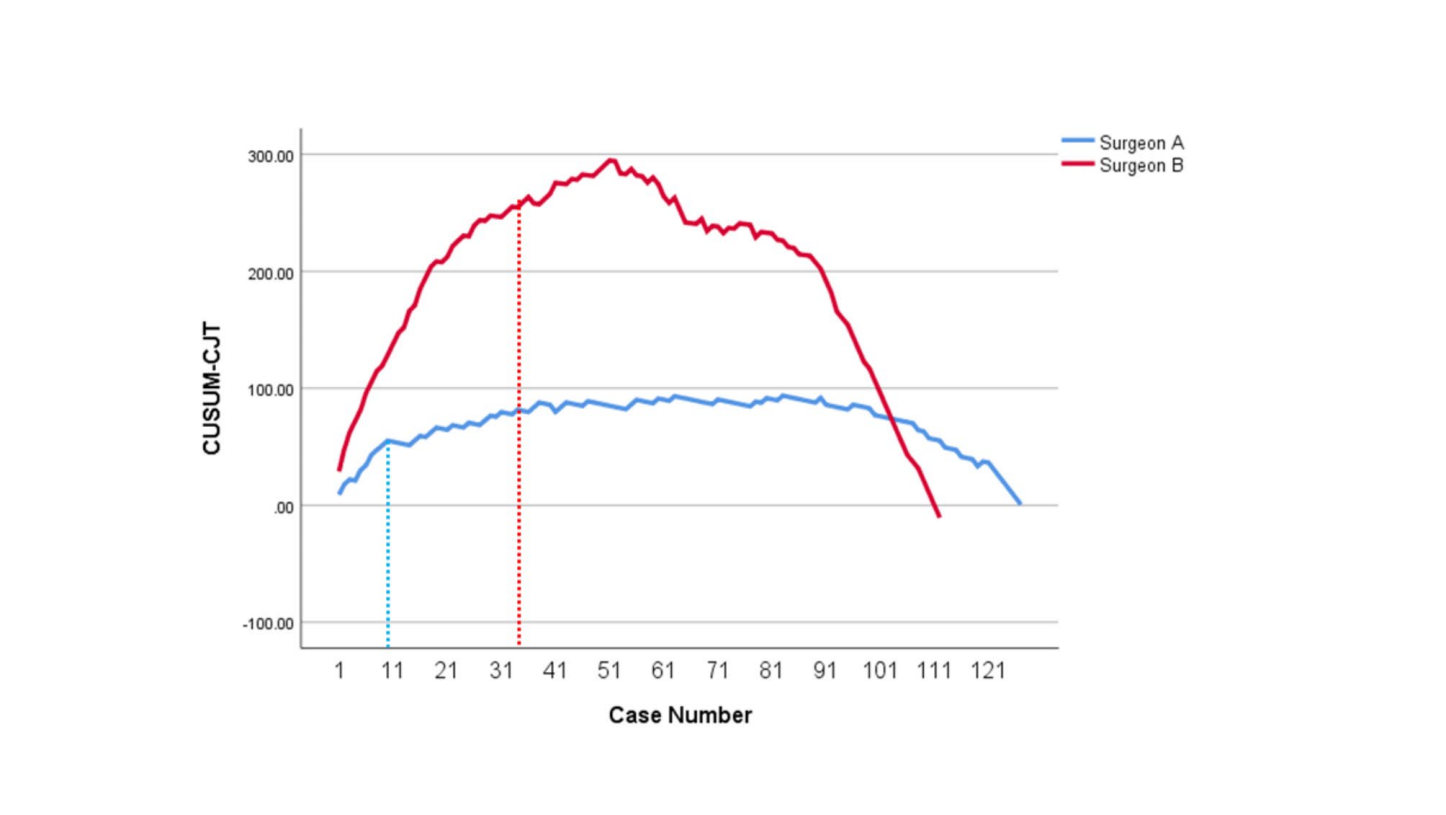
Cumulative sum graphs for choledochojejunostomy time (CUSUMCJT) of Surgeon A and Surgeon B

**Fig. 4:**
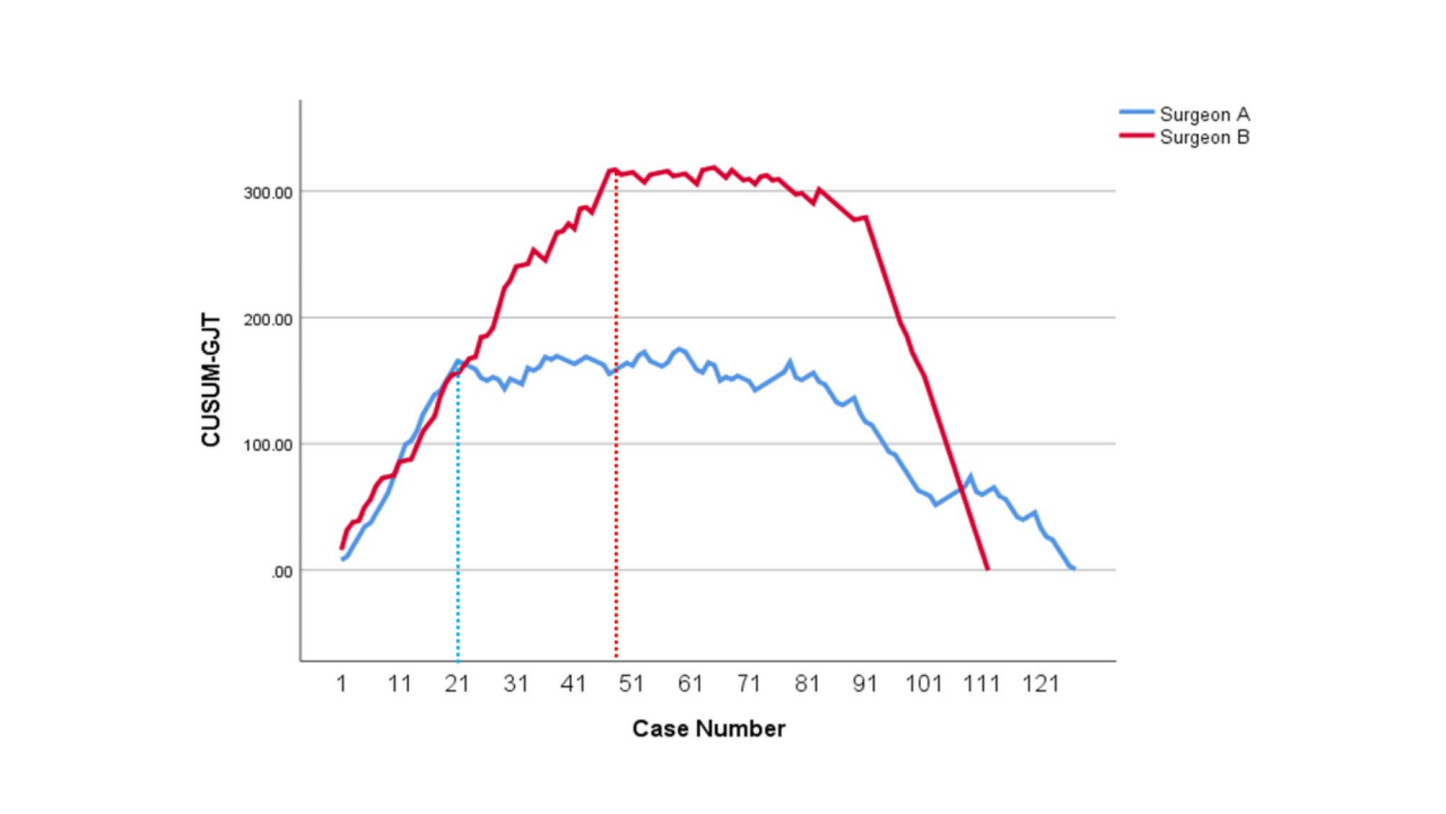
Cumulative sum graphs for gastrojejunostomy time (CUSUMGJT) of Surgeon A and Surgeon B

The learning curves of CUSUM_OT_, CUSUM_PJT_, CUSUM_CJT_ and CUSUM_GJT_ for Surgeon A and Surgeon B were developed, respectively. CUSUM_OT_ curves (Fig. 1) were divided into three phases, of which the second phases (the technical plateau phases) were observed approximately at Case # 25–41 and Case #35–51, for Surgeon A and Surgeon B, respectively. Initial peak points for CUSUM_OT_ curves, at which operators began to achieve technical competence for TLPD, were Case #25 and Case #35 for Surgeon A and Surgeon B, respectively. Initial peak points of CUSUM_PJT_ curves (Fig. 2) for Surgeon A and Surgeon B were approximately at Case #15 and Case #31, respectively. Initial peak points of CUSUM_CJT_ curve (Fig. 3) for Surgeon A and Surgeon B were approximately at Case #11 and Case #31, respectively. Initial peak points of CUSUM_GJT_ curve (Fig. 4) for Surgeon A and Surgeon B were approximately at Case #21 and #51, respectively. This suggested that Surgeon A required to accomplish fewer TLPD cases than Surgeon B, in order to achieve technical competence for TLPD surgical procedure.

CUSUM_OT_ curves consisted of three individual phases, namely, ascending (Cases #1–24 for Surgeon A; Cases #1–34 for Surgeon B), plateau (Cases #25–41 for Surgeon A; Cases #35–51 for Surgeon B), and the descending phase (Cases #42–127 for Surgeon A; Cases #52–112 for Surgeon B). Intra-surgical / post-surgical datasets were comparatively analyzed using statistical methods. No statistically significant variations were observed within pancreatic texture and MPD dimensions, among all differing phases for both surgeons. Intra-surgical hemorrhage demonstrated a decreasing trend from Phase 1 to Phase 3 for both surgeons, which were statistically significant. Post-surgical major complication rates, together with ICU stay durations, demonstrated a decreasing trend from Phase 1 to Phase 3 for both surgeons, which were statistically significant. The repeat surgery cases had statistically significant variation among the 3 phases. Statistical profiles / dataset outcomes for comparative analyses across differing LPD learning curve phases (both surgeons) are depicted within Tables 3 and 4, respectively.

**Table 3:** Statistical characteristics and results of the comparison tests of different phases of the LPD learning curve of Surgeon A. a: Median of the parameter; #: Independent-samples t tests; *: Chi-square tests; +: Kruskal-Wallis H tests

Parameter	Phase 1(Case #1–24)	Phase 2(Case #25–31)		Phase 3(Case #32–127)	P
Intraoperative					
Pancreatic texture [n (%)]					0.873^*^
Soft pancreas	22(91.67%)		6(85.71%)	85(88.54%)	
Not-soft pancreas	2(8.33%)		1(14.29%)	11(11.46%)	
MPD size(cm) ^a^	0.3		0.3	0.3	0.711^+^
Blood loss(mL)^a^	550		300	300	0.011^+^
Transfusion [n (%)]	14(58.33%)		3(42.86%)	40(41.67%)	0.338^*^
Transfusion volume(mL)^a^	200		200	0	0.074^+^
Complications [n (%)]					
Pancreatic fistula (BL/A/B/C)	20(83.33%)/0/3/1		5(71.43%)/0/1/1	71(73.96%)/2/7/5	0.604
Delayed gastric emptying	1(4.17%)		0	2(2.08%)	0.763^*^
Postoperative hemorrhage	2(8.33%)		2(28.57%)	5(5.21%)	0.020^*^
Abdominal infection	1(4.17%)		1(14.28%)	11(11.46%)	0.435^*^
Clavien-Dindo classification [n (%)]					< 0.001^*^
Minor(Grade I and II)	11(45.83%)		4(57.14%)	21(21.87%)	
Major(Grade III to V)	8(33.33%)		1(14.28%)	7(7.29%)	
Reoperation [n (%)]	5(20.83%)		0	2(2.08%)	0.001^*^
Death in hospital [n (%)]	2(8.33%)		0	2(2.08%)	0.146^*^
Hospital stay (day)^a^	19		15	10	0.097^+^
ICU stay (day)^a^	2		0	0	0.014^+^

**Table 4:** Statistical characteristics and results of the comparison tests of different phases of the LPD learning curve of Surgeon B

Parameter	Phase 1(Case #1–34)	Phase 2(Case #35–41)	Phase 3(Case #42–112)	P
Intraoperative				
Pancreatic texture [n (%)]				0.958^*^
Soft pancreas97	29(85.29%)	6(85.71%)	62	
Not-soft pancreas15	5(14.71%)	1(14.29%)	9	
MPD size(cm) ^a^	0.3	0.3	0.3	0.651^+^
Blood loss(mL)^a^	400	400	300	0.025^+^
Transfusion [n (%)]	28(82.35%)	5(71.43%)	55(77.46%)	0.787
Transfusion volume(mL)^a^	200	200	0	0.149^+^
Complications [n (%)]				
Pancreatic fistula (BL/A/B/C)	30/1/1/2	4/1/2/0	47/5/8/1	0.227^*^
Delayed gastric emptying	3(8.82%)	0	3(4.23%)	0.555^*^
Postoperative hemorrhage	8(23.53%)	2(28.57%)	5(7.04%)	0.035^*^
Abdominal infection	2(5.89%)	1(14.28%)	17(23.94%)	0.075^*^
Clavien-Dindo classification [n (%)]				0.013^*^
Minor(Grade I and II)	21(61.76%)	4(57.14%)	20(28.17%)	
Major(Grade III to V)	8(23.53%)	2(28.57%)	24(33.80%)	
Reoperation [n (%)]	5(14.71%)	3(42.86%)	6(8.45%)	0.029^*^
Death in hospital [n (%)]	2(5.89%)	0	2(2.82%)	0.636^*^
Hospital stay (day)^a^	20	21	18	0.457^+^
ICU stay (day)^a^	2	0	0	0.003^+^

a: Median of the parameter; #: Independent-samples t tests; *: Chi-square tests; +: Kruskal-Wallis H tests

## Discussion

TLPD has been widely accepted and applied in many medical centers globally [23]. During the learning / application process for TLPD, our surgical team embarked upon the uncinate-process-first, novel-artery-first and inferior-posterior duodenal approach [24–26] while also following the principle of ‘no touch’ in every procedure, from the beginning of such surgery [27]. To overcome the learning period, this study preferred and recommended the surgical options of ‘easy and safe steps first’. Based on our experience of > 700 LPD cases between 2013 and 2020, the key points to achieve LPD proficiency highly depended upon the technical skills of other laparoscopic surgeries, abundant OPD experience and effective teamwork. This study considered hand-assisted LPD, TLPD, TLPD with total mesopancreas excision (TMpE), and TLPD with vessel reconstruction, as four technical marks. Surgical teams in our department gradually and successfully performed hand-assisted LPD and TLPD, aiming at accomplishing these procedures: TLPD with total mesopancreas excision (TMpE) and TLPD with vessel reconstruction, focusing on the precision and standardization of such procedures.

The required training period could be determined by learning curves for TLPD. Several studies suggested that a steep LPD learning curve - that impacts patient outcomes - could be positively affected by appropriate training, high-volume practice/institution, proficient mentorship and an experienced multi-disciplinary team [6–8, 12, 16–18, 28–30]. Differing results for LPD learning curves were presented from previous studies, based upon differing methods. By statistically analyzing clinical data related to LPD, the learning curve was presented to be a steep curve [28, 31]. Through pre-defining the learning periods, 5 out of 12 cases, 10 out of 30 cases, 50 out of 56 cases, 30–60 out of 120 cases, and 47 out of 473 cases were required to reach the turning point respectively - after which there was significant reduction of blood loss and operation time [7, 8, 17, 32, 33]. Through CUSUM methods, the learning curve for LPD revealed that 12–38 out of 57 cases, 21–30 out of 50 cases, 20–25 out of 50 cases, 34–65 cases out of 98 cases, 47 out of 119 cases, 41–100 cases out of 171 cases, 55 cases (for the first-generation surgeon) out of 500 cases and 40–104 out of 133 cases (a retrospective multicenter analysis of 1029 patients) were required for proficiency in LPD, respectively [9, 10, 12–16, 18]. In summary, initial peak for learning curves involving > 50 cases was mainly within the range of 40–50 cases [7–10, 12–18]. This study concluded that sample size was determined according to the summary of learning curve-related studies.

Concomitant to the rapid development of laparoscopic technology, laparoscopic platforms are widely used within the field of hepatobiliary and pancreatic surgery. Nearly all such procedures can be accomplished through laparoscopy, within large medical centers. Junior surgeons have reduced opportunities to commence practice on open-surgery procedures. Regarding LPD, which is the one of the most challenging surgical procedures, the confirmation of how a surgeon with no OPD experience would be competent enough to perform a TLPD, has crucial importance. In order to mitigate such an issue, this study aimed at probing the influence of FAE upon TLPD learning curve for an operator. This investigation also aimed to minimize such factor effects, possibly influencing TLPD performance across both groups. These factors included laparoscopic equipment, surgical team, patient characteristics, pancreas consistency and MPD diameter. Both junior surgeons in this study had at least 6-years’ working experience, and were competent in fundamental laparotomic / laparoscopic hepatobiliary surgical procedures. Regarding PD, both surgeons had a FAE approximating 30 OPD cases of OPD, respectively, though had minimal experience as OPD operators. This study aimed at exploring the influence of FAE in TLPD on the learning curve for an operator, together with technical competence for both surgeons could be referenced. The statistically valid comparative analyses in this study suggested that enhanced post-operative recovery together with increased quantities of harvested lymph nodes were found within TLPD cases performed by a surgeon having previous FAE for TLPD, when compared to TLPD cases performed by a surgeon without previous FAE for TLPD. To prevent PPH in patients with deteriorating abdominal infection, we chose to initiatively perform laparoscopic debridement within one week post-LPD. Although that would lead to a slightly high reoperation rate, which was around 5–10% of the two surgeons, it was much safer for the patients. TLPD learning curves for both surgeons showed that the surgeon with FAE required less TLPD cases(# 25–41 cases) to accomplish prowess, in comparison to the surgeon with no previous FAE for TLPD(# 35–51 cases), in order to achieve technical competence for TLPD. Intra-surgery datasets (blood loss) and post-surgery datasets (post-surgery major complication rates, ICU durations and repeated surgery) of the three phases on the learning curve showed a descending trend for both surgeons, which was consistent with the learning curve.

There were limitations to this study. Firstly, there was a lack of comparative analyses for long-term results. Secondly, only two surgeons were involved in this study. Additional surgeons with differing technical levels of laparotomic and laparoscopic hepatobiliary surgery procedures, together with larger cohort sizes should be involved in further studies, in order to better-assess the positive factors for accelerating TLPD learning curve.

In conclusion, TLPD was a feasible, safe and effective surgery when performed by second-generation TLPD surgeons. FAE in TLPD can accelerate the learning curve for TLPD within an operator - with safer surgical procedures and enhanced post-operative recovery. Training and studying in large-volume TLPD centers could be a safer and more efficient method to positively affect the learning curve and clinical outcomes for such patients.

## Data Availability

The datasets used and analyzed during the current study available from the corresponding author on reasonable request.
